# Liraglutide, a once-daily human GLP-1 analogue, added to a sulphonylurea over 26 weeks produces greater improvements in glycaemic and weight control compared with adding rosiglitazone or placebo in subjects with Type 2 diabetes (LEAD-1 SU)

**DOI:** 10.1111/j.1464-5491.2009.02666.x

**Published:** 2009-03

**Authors:** M Marre, J Shaw, M Brändle, W M W Bebakar, N A Kamaruddin, J Strand, M Zdravkovic, T D Le Thi, S Colagiuri

**Affiliations:** Service d’Endocrinologie Diabétologie Nutrition, Groupe Hospitalier Bichat—Claude BernardParis, France; * International Diabetes InstituteMelbourne, Australia; † Division of Endocrinology, Diabetes and OsteologyKantonsspital St Gallen, Switzerland; ‡ Department of Medicine, Hospital Universiti SainsMalaysia, Kelantan; § Department of Medicine, National University of Malaysia (UKM)Kuala Lumpur, Malaysia; ¶ Oulun DiakonissalaitosOulu, Finland; ** Novo Nordisk A/SBagsvaerd, Denmark; †† Institute of Obesity, Nutrition and Exercise, University of SydneyAustralia

**Keywords:** dipeptidyl peptidase-4, glucagon-like peptide-1 receptor agonist, incretin, insulinotropic, thiazolidinedione

## Abstract

**Aim:**

To compare the effects of combining liraglutide (0.6, 1.2 or 1.8 mg/day) or rosiglitazone 4 mg/day (all *n* ≥ 228) or placebo (*n* = 114) with glimepiride (2–4 mg/day) on glycaemic control, body weight and safety in Type 2 diabetes.

**Methods:**

In total, 1041 adults (mean ± sd), age 56 ± 10 years, weight 82 ± 17 kg and glycated haemoglobin (HbA_1c_) 8.4 ± 1.0% at 116 sites in 21 countries were stratified based on previous oral glucose-lowering mono : combination therapies (30 : 70%) to participate in a five-arm, 26-week, double-dummy, randomized study.

**Results:**

Liraglutide (1.2 or 1.8 mg) produced greater reductions in HbA_1c_ from baseline, (−1.1%, baseline 8.5%) compared with placebo (+0.2%, *P* < 0.0001, baseline 8.4%) or rosiglitazone (−0.4%, *P* < 0.0001, baseline 8.4%) when added to glimepiride. Liraglutide 0.6 mg was less effective (−0.6%, baseline 8.4%). Fasting plasma glucose decreased by week 2, with a 1.6 mmol/l decrease from baseline at week 26 with liraglutide 1.2 mg (baseline 9.8 mmol/l) or 1.8 mg (baseline 9.7 mmol/l) compared with a 0.9 mmol/l increase (placebo, *P* < 0.0001, baseline 9.5 mmol/l) or 1.0 mmol/l decrease (rosiglitazone, *P* < 0.006, baseline 9.9 mmol/l). Decreases in postprandial plasma glucose from baseline were greater with liraglutide 1.2 or 1.8 mg [−2.5 to −2.7 mmol/l (baseline 12.9 mmol/l for both)] compared with placebo (−0.4 mmol/l, *P* < 0.0001, baseline 12.7 mmol/l) or rosiglitazone (−1.8 mmol/l, *P* < 0.05, baseline 13.0 mmol/l). Changes in body weight with liraglutide 1.8 mg (−0.2 kg, baseline 83.0 kg), 1.2 mg (+0.3 kg, baseline 80.0 kg) or placebo (−0.1 kg, baseline 81.9 kg) were less than with rosiglitazone (+2.1 kg, *P* < 0.0001, baseline 80.6 kg). Main adverse events for all treatments were minor hypoglycaemia (< 10%), nausea (< 11%), vomiting (< 5%) and diarrhoea (< 8%).

**Conclusions:**

Liraglutide added to glimepiride was well tolerated and provided improved glycaemic control and favourable weight profile.

## Introduction

Most drugs that target Type 2 diabetes (T2D) also cause weight gain or hypoglycaemia, or both, with the risk increasing with combination therapy. Glucagon-like peptide-1 (GLP-1)-based therapies stimulate insulin secretion and reduce glucagon secretion only during hyperglycaemia. GLP-1 also slows gastric emptying and reduces appetite [1].

Although American Diabetes Association (ADA)/European Association for the Study of Diabetes (EASD) guidelines recommend lifestyle and metformin as initial therapy for T2D [2], sulphonylureas are used widely, particularly when metformin or thiazolidinediones are not tolerated. Glycaemic control eventually deteriorates with sulphonylureas while hypoglycaemia and weight gain are common [3]. Incretin therapy improves glycaemic control with low hypoglycaemic risk, while delayed gastric emptying and reduced appetite can reduce weight [1,4].

Liraglutide is a once-daily human GLP-1 analogue with 97% linear amino-acid sequence homology to human GLP-1 [5] and half-life of 13 h after subcutaneous administration that produces 24-h blood glucose control [6]. Liraglutide monotherapy for 14 weeks reduced glycated haemoglobin (HbA_1c_) by 1.7% and fasting plasma glucose (FPG) by 3.4 mmol/l without causing hypoglycaemia, along with weight loss (∼3 kg) compared with placebo [7]. Improvements in pancreatic B-cell function [7–9] and blood pressure [7], along with decreased glucagon secretion [7,10], also occurred. As part of the phase 3 programme [the Liraglutide Effect and Action in Diabetes (LEAD) programme] with liraglutide in > 4000 subjects with T2D as monotherapy or in combination therapy, this 26-week trial examined liraglutide plus glimepiride compared with either placebo or rosiglitazone added to glimepiride on glycaemic control and body weight.

## Subjects and methods

### Study participants

Inclusion criteria: T2D treated with oral glucose-lowering agents (OGLAs) for ≥ 3 months; 18–80 years of age; HbA_1c_ 7.0–11.0% (previous OGLA monotherapy) or 7.0–10.0% (previous OGLA combination therapy); body mass index (BMI) ≤ 45.0 kg/m^2^. Exclusion criteria: used insulin within 3 months, impaired liver or renal function, uncontrolled hypertension (≥ 180/100 mmHg), cancer or used any drugs apart from OGLAs likely to affect glucose concentrations. Subjects provided written informed consent. The study was conducted in accordance with good clinical practice guidelines and approved by independent ethics committees.

### Study design

**FIGURE 1:**
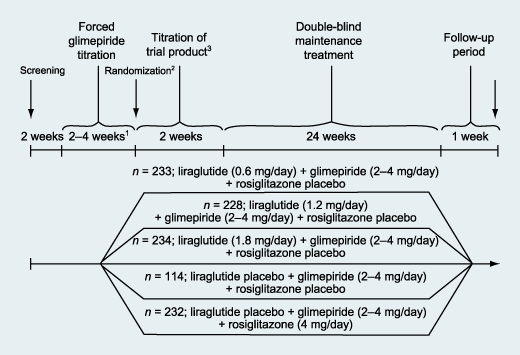
Overview of trial design and treatment arms.

The study was a 26-week, double-blind, double-dummy, randomized, active-control, five-armed parallel (116 sites in 21 countries, primarily Europe and Asia) trial enrolling 1041 subjects (1–37 subjects per centre), all receiving glimepiride (2–4 mg/day) in combination with (Fig. 1):

- one of three liraglutide doses [0.6, 1.2 or 1.8 mg, injected subcutaneously (Novo Nordisk, Bagsvaerd, Denmark) and rosiglitazone placebo];
- liraglutide placebo and rosiglitazone placebo;
- liraglutide placebo and rosiglitazone 4 mg/day (rosiglitazone; Avandia™; GlaxoSmithKline, London, UK).

The doses of rosiglitazone and glimepiride used were determined by the highest doses approved in all participating counties. After discontinuing previous OGLAs except glimepiride, separate 2-week titration and maintenance periods with glimepiride (open-label) preceded randomization (Fig. 1). Subjects were stratified according to previous treatment (monotherapy or combination therapy). After randomization, 2-week treatment titration and 24-week treatment (maintenance) phases (Fig. 1) were completed. Liraglutide was up-titrated weekly in 0.6-mg increments until allocated doses were reached. Glimepiride could be adjusted between 2 and 4 mg/day in case of hypoglycaemia or other adverse events (AEs), while other drug doses were fixed. Liraglutide (active and placebo) was supplied in 3-ml pre-filled pens with 31G needles (Novo Nordisk). Subjects were encouraged to inject liraglutide into the upper arm, thigh or abdomen at the same time each day. Rosiglitazone and glimepiride were taken in the morning or with the first meal.

### Study measurements

#### Efficacy

The primary endpoint was change from baseline HbA_1c_ after 26 weeks of treatment. Secondary endpoints included: percentages of subjects reaching HbA_1c_ (< 7.0%, ≤ 6.5%), FPG (5.0 to ≤ 7.2 mmol/l) and postprandial plasma glucose (PPG; 10.0 mmol/l) targets [11–13]; changes in body weight, FPG, mean PPG, indices of pancreatic B-cell function [pro-insulin : insulin ratio and homeostasis model assessment (HOMA)-B], HOMA-insulin resistance (HOMA-IR) and blood pressure (BP).

HbA_1c_ was measured centrally (MDS Pharma Services, King of Prussia, PA, USA) by high performance liquid chromatography while plasma glucose (PG) was self-measured using MediSense® glucose meters (Abbott Diagnostics Inc., Abbott Park, IL, USA). Insulin and C-peptide were measured by chemiluminescence, proinsulin by ELISA, while glucagon was measured in aprotinin-treated plasma by radioimmunoassay. The proinsulin : insulin ratio was calculated from fasting insulin and fasting proinsulin. HOMA-B and HOMA-IR were both calculated from FPG and fasting insulin. Samples measured centrally were collected and transported according to detailed procedures in the MDS Pharma Services manual. Samples stored at ambient temperature were shipped by courier to the central laboratory on the same day as collection, while frozen samples were shipped every 3 weeks.

#### Safety

Safety variables included hypoglycaemic episodes based on PG levels (< 3.1 mmol/l), liraglutide antibodies including cross-reacting and neutralizing antibodies, tolerability (gastrointestinal complaints) and pulse. AEs, vital signs, electrocardiogram (ECG), biochemical and haematology measures including calcitonin were also monitored. Self-treated hypoglycaemic episodes were classified as minor, while those requiring third-party assistance were considered major. Serum antibodies against liraglutide were measured by radioimmunoprecipitation assay.

### Statistical analyses

All efficacy and safety analyses were based on intent-to-treat criteria, defined as subjects who were exposed to ≥ 1 dose of trial product(s). Efficacy endpoints were analysed by ancova with treatment, country and previous glucose-lowering treatment as fixed effects and baseline values as covariates. Missing data were imputed by last observation carried forward (LOCF). Sample size calculations were based on predicted HbA_1c_ and body weight after trial completion. As the three liraglutide + glimepiride groups were to be compared with both rosiglitazone + glimepiride and glimepiride monotherapy, two calculations were performed. These sample size calculations assumed a standard deviation of 1.2% of HbA_1c_, the non-inferiority/superiority margin vs. active control was set to 0.4% and the difference to detect (superiority vs. placebo) was set to 0.5%. For body weight, a coefficient of variation of 3% (based on phase 2a trials for liraglutide) and a difference to detect of 3% were assumed. A combined power (calculated as the product of the marginal powers for HbA_1c_ and body weight) of at least 85% was required. These calculations indicated that at least 168 and 81 patients completing the study would be needed for the combination and glimepiride monotherapy groups, respectively. Assuming a drop-out rate of 25%, targets for randomization were 228 in each of the combination therapy groups and 114 in the placebo group (total *n* = 1026).

To protect against Type 1 errors, HbA_1c_ was analysed using hierarchical testing for descending doses of liraglutide. First, superiority of liraglutide 1.8 mg to placebo was tested and, only if superior to placebo, non-inferiority to rosiglitazone was tested. If non-inferiority was obtained, superiority to rosiglitazone for liraglutide 1.8 mg was tested and superiority to placebo for liraglutide 1.2 mg was tested. If superiority was confirmed, non-inferiority to rosiglitazone would be tested and so on (i.e. testing sequence was stopped when hypotheses could not be rejected). Superiority was concluded when upper limits of two-sided 95% confidence intervals (CIs) for treatment differences were below 0%; non-inferiority was concluded if these values were < 0.4%; for secondary endpoints, Type 1 errors were controlled by estimating simultaneous CIs using Dunnett's method.

Proportions of subjects achieving HbA_1c_ (HbA_1c_ < 7.0%, and ≤ 6.5%) and FPG (5.0 ≤ FPG ≤ 7.2 mmol/l) targets [13] were compared between treatments using logistic regression with allocated treatment and baseline values as covariates. Chi-square analyses assessed differences in treatments for percentages of subjects achieving no, one, two or three PPG values < 10 mmol/l [13]. Hypoglycaemic episodes were analysed under the assumption that number per subject were negatively binomially distributed using a generalized linear model, including treatment and country as fixed effects. Other safety data were compared by descriptive statistics. Values for descriptive statistics are expressed as means ± sd, while ancova results are expressed as least square means ± SEM or with 95% CI unless otherwise noted. Significance levels were set to 5% for two-sided tests and 2.5% for one-sided tests.

## Results

### Disposition and demographics

**FIGURE 2:**
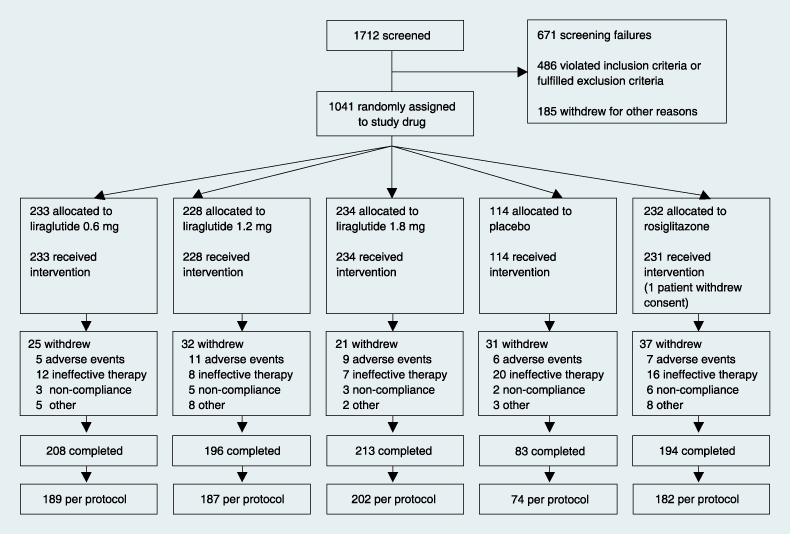
Flow of patients through the study.

The treatment groups were well balanced (Table 1). Of 1712 subjects screened, 1041 were randomized and 1040 were exposed to trial drugs; 147 subjects (14.1%) withdrew (Fig. 2). Withdrawals were higher with placebo (27%) and rosiglitazone treatment (16%) compared with liraglutide 0.6 mg (11%), liraglutide 1.2 mg (14%) and liraglutide 1.8 mg (9%) treatment. Thirty-eight subjects (3.7%) withdrew as a result of AEs (Fig. 2).

**Table 1:** Demographic characteristics of study participants

	Liraglutide 0.6 mg (*n* = 233)	Liraglutide 1.2 mg (*n* = 228)	Liraglutide 1.8 mg (*n* = 234)	Placebo (*n* = 114)	Rosiglitazone (*n* = 232)
Male : female (%)	54 : 46	45 : 55	53 : 47	47 : 53	47 : 53
Age (years)	55.7 ± 9.9	57.7 ± 9.0	55.6 ± 10.0	54.7 ± 10.0	56.0 ± 9.8
Duration of diabetes (years)	6.5 (4.0,10.2)	6.7 (4.0,10.7)	6.5 (3.7,10.5)	6.5 (4.5,10.6)	6.6 (4.3,10.7)
Previous on mono : combi (%)	30 : 70	31 : 69	27 : 73	32 : 68	32 : 68
FPG (mmol/l)	10.0 ± 2.4	9.8 ± 2.7	9.7 ± 2.4	9.5 ± 2.0	9.9 ± 2.5
HbA_1c_ (%)	8.4 ± 1.0	8.5 ± 1.1	8.5 ± 0.9	8.4 ± 1.0	8.4 ± 1.0
Diabetic retinopathy (%)	17.2	14.9	12.0	13.2	16.4
Hypertension (%)	69.1	68.0	69.7	64.9	66.8
BMI (kg/m^2^)	30.0 ± 5.0	29.8 ± 5.1	30.0 ± 5.1	30.3 ± 5.4	29.4 ± 4.8
Weight (kg)	82.6 ± 17.7	80.0 ± 17.1	83.0 ± 18.1	81.9 ± 17.1	80.6 ± 17.0
Systolic blood pressure (mmHg)	131 ± 16	133 ± 15	132 ± 16	131 ± 15.3	133 ± 15

Data are mean ± sd and percentages, except for duration of diabetes, where data are median, 25th and 75th percentile.

BMI, body mass index; FPG, fasting plasma glucose; HbA_1c_, glycated haemoglobin; mono : combi, previous treatment with either monotherapy or combination therapy; sd, standard deviation.

### Efficacy

#### HbA_1c_

**FIGURE 3:**
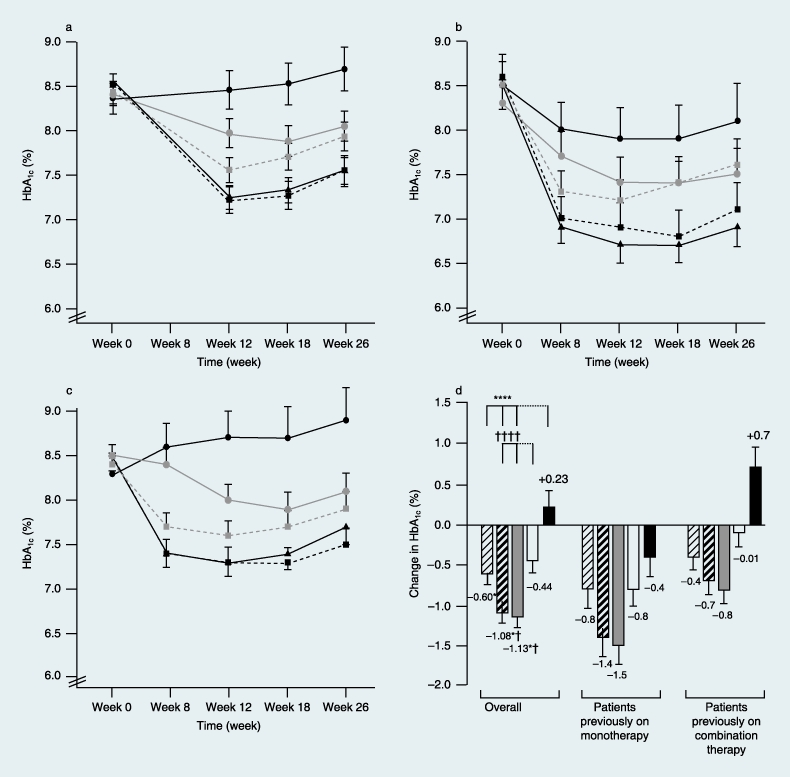
Mean glycated haemoglobin (HbA_1c_) by treatment and week (intent-to-treat population with last observation carried forward): (a) overall population; (b) previously on monotherapy; or (c) previously on combination therapy; (d) mean changes in HbA_1c_ from baseline after 26 weeks of treatment. Keys: (a–c) liraglutide 0.6 mg: grey dotted line with squares; liraglutide 1.2 mg: black solid line with triangles; liraglutide 1.8 mg: black dotted line with squares; rosiglitazone: grey solid line with circles; placebo: black solid line with circles. (d) liraglutide 0.6 mg: black stripes on white; liraglutide 1.2 mg: white stripes on black, liraglutide 1.8 mg: grey tint; rosiglitazone: white; placebo: black. ****P < 0.0001 compared with placebo; ††††*P* < 0.0001 compared with rosiglitazone.

HbA_1c_ decreased rapidly with all doses of liraglutide when added to glimepiride compared with either rosiglitazone or placebo (i.e. glimepiride monotherapy), irrespective of previous therapy. The greatest decreases occurred with liraglutide 1.2 and 1.8 mg (Fig. 3a–c). After 26 weeks, HbA_1c_ decreased by 1.1% from baseline (primary endpoint) with either liraglutide 1.2 or 1.8 mg, respectively, compared with either placebo (+0.2%) or rosiglitazone (−0.4%) (Fig. 3d). Estimated treatment differences and 95% CIs to placebo were: liraglutide 1.8 mg: −1.4% (1.6, −1.1); liraglutide 1.2 mg: −1.3% (1.5, −1.1); liraglutide 0.6 mg: −0.8% (−1.1, −0.6); rosiglitazone: −0.7% (−0.9, −0.4). All liraglutide doses were superior to placebo (*P* < 0.0001), while the two higher liraglutide doses were superior to rosiglitazone (*P* < 0.0001). Liraglutide 0.6 mg was non-inferior to rosiglitazone. Rosiglitazone also was superior to placebo (*P* < 0.0001).

HbA_1c_ decreases were greater for subjects who entered from monotherapy compared with combination therapy (Fig. 3d). However, because the increase with placebo was higher for individuals entering on combination therapy (0.7 vs. 0.23%), the differences between treatment groups in favour of liraglutide were similar irrespective of whether subjects were treated previously with monotherapy or combination therapy. Neither age, gender nor BMI affected these trends.

#### Percentage reaching an HbA_1c_ < 7.0% and ≤ 6.5%

**FIGURE 4:**
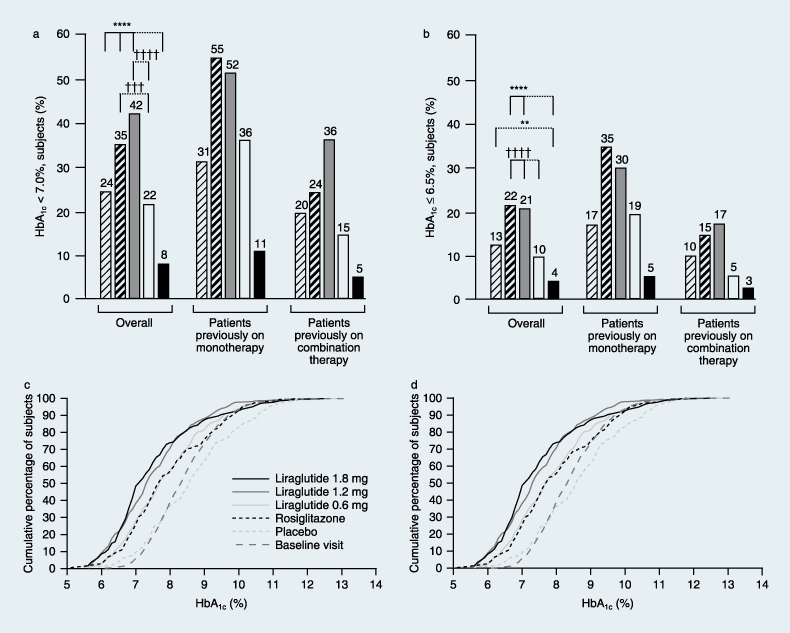
Subjects achieving specified glycated haemoglobin (HbA_1c_) levels: (a) percentage reaching HbA_1c_ < 7.0% (American Diabetes Association/European Association for the Study of Diabetes target); (b) percentage reaching HbA_1c_ < 6.5% (International Diabetes Federation/American Association of Clinical Endocrinologists targets); (c) cumulative distribution of HbA_1c_ at 26 weeks for the intent-to-treat (ITT) population; and (d) for the ITT last observation carried forward (LOCF) population. Keys: (a, b) liraglutide 0.6 mg: black stripes on white; liraglutide 1.2 mg: white stripes on black, liraglutide 1.8 mg: grey tint; rosiglitazone: white; placebo: black. (c, d) liraglutide 0.6 mg: pale grey solid line; liraglutide 1.2 mg: grey solid line, liraglutide 1.8 mg: black solid line; rosiglitazone: dotted black line; placebo: dotted grey line; baseline visit: long dashed black line. ****P < 0.0001 or **P < 0.01 compared with placebo; ††††*P* < 0.0001 or †††*P* = 0.0005 compared with rosiglitazone.

The percentage of subjects reaching ADA [2] and International Diabetes Federation (IDF)/American Association of Clinical Endocrinologists (AACE) [11,12] treatment HbA_1c_ goals with liraglutide was dose dependent (Fig. 4). At week 26, 42% and 21% of subjects treated with liraglutide 1.8 mg reached an HbA_1c_ < 7.0% and ≤ 6.5%, respectively, compared with 8% and 4% for placebo (Fig. 4). The estimated proportion of subjects treated with either liraglutide 1.2 or 1.8 mg reaching ADA/EASD and IDF/AACE HbA_1c_ targets was substantially greater compared with either placebo (*P* < 0.0001) or rosiglitazone (Fig. 4; *P* ≤ 0.0003), with more patients reaching < 7.0% with liraglutide 1.8 mg compared with 1.2 mg (*P* = 0.018).

#### Fasting plasma glucose

**FIGURE 5:**
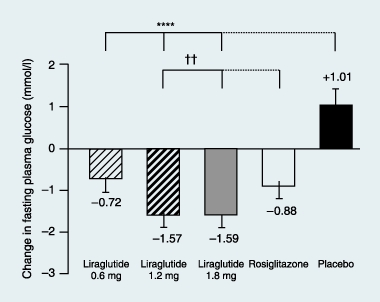
Mean changes from baseline in fasting plasma glucose after 26 weeks of treatment. ****P < 0.0001 compared with placebo; ††*P* < 0.01 compared with rosiglitazone.

By week 2, subjects treated with liraglutide had rapid and larger decreases in FPG vs. comparator treatment. At week 26, all doses of liraglutide decreased FPG more than did placebo (Fig. 5; *P* < 0.0001), while only liraglutide 1.2 or 1.8 mg produced greater reductions than rosiglitazone. FPG treatment differences to placebo were 1.7 mmol/l for liraglutide 0.6 mg and 2.6 mmol/l for both liraglutide 1.2 and 1.8 mg. An 0.7-mmol/l greater reduction in FPG was achieved with either liraglutide 1.2 or 1.8 mg compared with rosiglitazone (*P* ≤ 0.006) after 26 weeks.

The percentage of subjects achieving FPG values between 5.0 mmol/l and ≤ 7.2 mmol/l (ADA target) after 26 weeks was higher with liraglutide: 0.6 mg (19%; *P* = 0.002); 1.2 mg (37%; *P* < 0.001); and 1.8 mg (38%;*P* < 0.001) compared with placebo (7%). The liraglutide 1.2 and 1.8 mg treatment groups also had more subjects achieving the same FPG target at end of treatment compared with rosiglitazone (26%) (*P* = 0.007 and *P* = 0.01, respectively).

#### Postprandial plasma glucose

PPG was reduced similarly after each meal. The greatest reductions in mean PPG values from baseline (average of values obtained 90 min after breakfast, lunch and evening meal) occurred with liraglutide 1.2 mg (2.5 mmol/l) and liraglutide 1.8 mg (2.7 mmol/l). By comparison, the reduction from baseline in mean PPG values was 1.8 mmol/l for rosiglitazone and liraglutide 0.6 mg and 0.4 mmol/l for placebo. Treatment differences for PPG were greater with all doses of liraglutide compared with placebo (1.5–2.4 mmol/l; *P* < 0.0001) and greater with liraglutide 1.2 mg (0.64 mmol/l; *P* = 0.043) and 1.8 mg (0.87 mmol/l;*P* = 0.0022) compared with rosiglitazone.

#### PPG measurements < 10.0 mmol/l

The percentage of subjects with one, two or three PPG measurements < 10.0 mmol/l (ADA target) were greater for all doses of liraglutide compared with placebo (*P* < 0.05) but not rosiglitazone.

### Body weight

**FIGURE 6:**
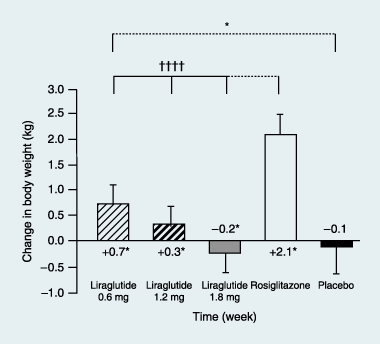
Mean changes in body weight from baseline after 26 weeks of treatment. *P < 0.05 compared with placebo; ††††*P* < 0.0001 compared with rosiglitazone.

Mean weight at baseline was 81.6 kg. Mean reductions in weight from baseline to end of treatment were 0.2 kg with liraglutide 1.8 mg and 0.1 kg with placebo treatment, while increases occurred with either liraglutide 0.6 mg (0.7 kg), liraglutide 1.2 mg (0.3 kg) or rosiglitazone (2.1 kg) (Fig. 6). Unlike rosiglitazone, weight did not increase substantially with liraglutide and the differences between rosiglitazone and liraglutide were statistically significant (−2.3 to −1.4 kg; *P* < 0.0001), although there were no significant differences compared with placebo. Gender appeared to have no influence on the results, as indicated when added as a fixed effect in the ancova model.

### Indices of pancreatic B-cell function and insulin resistance

Reductions in the proinsulin : insulin ratio were greater with both liraglutide 1.2 and 1.8 mg compared with either rosiglitazone or placebo (Table 2; *P* ≤ 0.02). HOMA-B increased with liraglutide (1.8 or 1.2 mg) compared with rosiglitazone (*P* < 0.05), while this increase was only different to placebo with liraglutide 1.2 mg (*P* = 0.01) and not liraglutide 1.8 mg (*P* = 0.051). There were no significant differences between treatments for HOMA-IR.

**Table 2:** Selected indices of pancreatic B-cell function

Variable	Treatment	Baseline	Week 26 (LOCF)	Least square difference from placebo (95% CI)	Least square difference from rosiglitazone (95% CI)
Proinsulin : insulin ratio	Liraglutide 0.6 mg	0.42 ± 0.22	0.38 ± 0.24	−0.05 (−0.11; 0.00)	−0.02 (−0.06; 0.03)
	Liraglutide 1.2 mg	0.45 ± 0.31	0.33 ± 0.20	−0.10 (−0.16; −0.05)†	−0.07 (−0.11; −0.02)*
	Liraglutide 1.8 mg	0.48 ± 0.33	0.36 ± 0.20	−0.09 (−0.15; −0.03)*	−0.05 (−0.10; −0.01)*
	Placebo	0.44 ± 0.27	0.46 ± 0.29		
	Rosiglitazone	0.45 ± 0.29	0.40 ± 0.20		
HOMA-B (%)	Liraglutide 0.6 mg	51 ± 43.3	70 ± 88.6	15 (−19.10; 49.0)	11 (−16.7; 39.0)
	Liraglutide 1.2 mg	71 ± 254.3	99 ± 184.3	43 (8.10; 76.9)*	39 (10.3; 67.0)*
	Liraglutide 1.8 mg	56 ± 84.6	91 ± 108.2	34 (−0.23; 68.5)	30 (2.00; 58.6)*
	Placebo	56 ± 103.3	52 ± 107.3		
	Rosiglitazone	46 ± 36.2	59 ± 63.3		

* *P*≤ 0.05;

† *P* < 0.0001.

CI, confidence interval; HOMA, homeostatis model assessment; LOCF, last observation carried forward.

### Blood pressure and pulse

Although decreases in systolic blood pressure occurred with either liraglutide 1.2 or 1.8 mg (2.6–2.8 mmHg), they were not significantly different from placebo or rosiglitazone (0.9–2.3 mmHg). Reductions in diastolic blood pressure also occurred with all treatments (0.7–1.4 mmHg), with no significant differences between treatments. Pulse increases above baseline ranged from 2 to 4 beats/min with the three doses of liraglutide and 1 beat/min with rosiglitazone, while pulse decreased by 1 beat/min with placebo. Changes in pulse for all doses of liraglutide were significant vs. placebo (*P* ≤ 0.002). This also was true with either liraglutide 1.8 or 1.2 mg compared with rosiglitazone (*P* < 0.01).

### Safety

The most common treatment-emergent AEs that were considered by investigators to be either possibly or probably related to liraglutide were gastrointestinal (diarrhoea, nausea, dyspepsia and constipation) and nervous system disorders (headache and dizziness), particularly during the first 4 weeks. Nausea was highest with liraglutide 1.2 mg (10.5%) and lowest with placebo (1.8%). Vomiting (4.4%) and diarrhoea (7.9%) were also higher with liraglutide 1.2 mg. Withdrawals because of nausea ranged from 0.9–2.2%, vomiting 0.4–0.9% and diarrhoea 0–1.3%.

**FIGURE 7:**
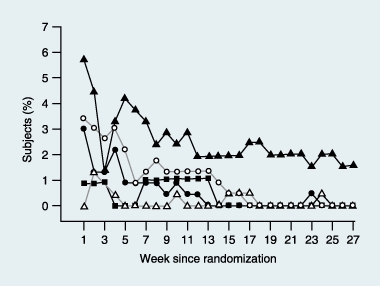
Percentage of subjects experiencing nausea over the course of the study. Key: liraglutide 0.6 mg with glimepiride: black line with filled circles; liraglutide 1.2 mg with glimepiride: black line with filled triangles; liraglutide 1.8 mg with glimepiride: grey line with hollow circles; glimepiride grey lines with filled squares; rosiglitazone and glimepiride: grey line with hollow triangles.

Nausea was more common with liraglutide compared with placebo and rosiglitazone, particularly during the first 4 weeks (Fig. 7). Frequency of nausea was less in the liraglutide 0.6 mg treatment group compared with the higher doses of liraglutide. Generally, the occurrence of nausea dissipated from 4 to 26 weeks of treatment in all groups using liraglutide (Fig. 7).

The incidence of serious AEs ranged between 3 and 5%: placebo (3%), rosiglitazone (3%), liraglutide 0.6 mg (3%), liraglutide 1.2 mg (4%) and liraglutide 1.8 mg (5%). Most treatment-emergent serious AEs were judged by investigators to be unlikely to be related to trial products. No deaths were reported during the trial. One subject developed chronic pancreatitis whilst taking liraglutide 0.6 mg; the person had no reported previous history of pancreatitis. The subject continued on liraglutide therapy and completed the trial. At screening, five patients had been previously diagnosed with pancreatitis. As pancreatitis was not an exclusion criterion, these patients were randomized as follows: one to liraglutide 0.6 mg, one to liraglutide 1.2 mg, two to liraglutide 1.8 mg and one to rosiglitazone + glimepiride. All five patients completed the trial without reporting pancreatitis as an adverse event.

Hypoglycaemia was infrequent with all treatments. One major hypoglycaemic episode (self-measured blood glucose = 3.0 mmol/l) occurred 9 days after treatment started in a subject receiving liraglutide 1.8 mg in combination with glimepiride. Although medical assistance was not needed, the subject required third-party assistance. The investigator judged the episode as likely to be related to glimepiride and reduced the dose from 4 to 3 mg after the incident.

Minor hypoglycaemia occurred in < 10% of subjects for any treatment. The proportion of subjects experiencing minor hypoglycaemia during the trial was lowest with placebo (i.e. glimepiride monotherapy 2.6%; 0.17 events/subject-year), comparable with liraglutide 0.6 mg (5.2%, 0.17 events/subject-year) and rosiglitazone (4.3%, 0.12 events/subject-year) groups and similar between the liraglutide 1.2 mg (9.2%, 0.51 events/subject-year) and liraglutide 1.8 mg (8.1%, 0.47 events/subject-year) treatment groups. Incidence was higher with liraglutide 1.2 mg (*P* = 0.0024) and 1.8 mg (*P* = 0.0065) compared with rosiglitazone and liraglutide 1.2 mg compared with placebo (*P* = 0.048), occurring in the setting of lower mean HbA_1c_ values.

Antibodies to liraglutide were found in 9–13% of subjects treated with liraglutide. No significant effects of these antibodies on HbA_1c_ were found in pooled analyses of four trials including the current study. There were no clinically relevant changes in ophthalmoscopy, biochemistry, urinalysis, haematology or ECG assessments. No significant differences in calcitonin were found between the three groups treated with liraglutide when compared with either placebo or rosiglitazone at the end of the trial at week 26.

## Discussion

Treatment with liraglutide plus glimepiride was superior to glimepiride monotherapy at all doses of liraglutide and superior to rosiglitazone plus glimepiride for the two higher liraglutide doses for improving HbA_1c_. Similar findings for reductions in FPG and PPG highlight improved 24-h glucose control with once-daily liraglutide, with substantially more subjects reaching glycaemic targets, particularly with liraglutide 1.8 mg. Improvements in pancreatic B-cell function were larger with liraglutide 1.2 and 1.8 mg compared with rosiglitazone. Liraglutide was well tolerated and occurrence of gastrointestinal AEs was low overall, particularly after week 4.

Although rates of hypoglycaemia were low in all treatment groups (< 10%), minor hypoglycaemic events occurred more often in patients treated with glimepiride plus liraglutide 1.2 or 1.8 mg than with glimepiride alone. It should be noted, however, that patients treated with liraglutide 1.2 or 1.8 mg achieved a lower HbA_1c_ than those receiving glimepiride monotherapy. At lower HbA_1c_ levels, sulphonylureas are known to elicit hypoglycaemia more readily than at higher levels. In clinical practice it may be possible to reduce the dose of sulphonylurea (when used with liraglutide) to minimize risk of hypoglycaemia and maintain HbA_1c_improvements.

Although weight effects were modest, liraglutide produced more favourable weight effects compared with rosiglitazone, which produced substantial weight gain. In other studies with liraglutide, subjects adding a 1.8-mg dose to metformin lost 2.8 kg [14], while those adding both metformin and glimepiride lost 1.8 kg compared with placebo [15] (both over 26 weeks) and those on liraglutide monotherapy (1.8 mg) lost 2.45 kg over 52 weeks [16]. In our study, because sulphonylureas usually cause weight gain, inclusion or optimization of glimepiride but not metformin may have mitigated the weight benefits typically associated with liraglutide. Lack of weight effects could be secondary to lower baseline body weight, withdrawal of previous metformin treatment or defensive snacking to minimize risk of hypoglycaemia.

It might have been expected that the greater weight gain with rosiglitazone compared with liraglutide 1.8 mg would be associated with a concurrent increase in insulin resistance with rosiglitazone. The absence of this effect could reflect the insulin-sensitizing nature of rosiglitazone. Improvements in pancreatic B-cell function associated with liraglutide are consistent with other studies [7–9].

Study strengths include inclusion of both placebo and active (rosiglitazone) comparators and that OGLAs were optimized (not maximized) before randomization to minimize risk of hypoglycaemia. Limitations of the study include short duration of the trial and restriction on glimepiride and rosiglitazone in some countries that precluded maximal dosing.

The impact of using other GLP-1-based treatments [such as exenatide, or the dipeptidyl peptidase-4 (DPP-4) inhibitor, sitagliptin] with sulphonylureas in subjects with T2D has been studied. In a 30-week American trial where exenatide twice a day was added to sulphonylureas, HbA_1c_ was reduced by 0.46% from baseline with 5 µg and 0.86% with 10 µg [17] compared with 1.1% with liraglutide 1.8 or 1.2 mg. This reduction in HbA_1c_ with liraglutide is consistent with other LEAD trials investigating liraglutide as monotherapy or in combination with various OGLA drugs. In these trials, HbA_1c_ was reduced by 1–1.5%[14,16,18–20]. Reductions in FPG with exenatide were 0.3 and 0.6 mmol/l from baseline with 5 µg and 10 µg, respectively, compared with 1.4 mmol/l with liraglutide 1.8 mg; weight loss of 1.6 kg occurred with exenatide 10 µg compared with 0.2 kg for liraglutide 1.8 mg [17]. Differences in weight effects may be as a result of lower baseline weight in this trial (82 kg) compared with exenatide (96 kg) and discontinuation of previous metformin therapy, unlike the exenatide trial where exenatide was added to previous sulphonylurea monotherapy [17]. Other large-scale trials with liraglutide in combination with sulphonylureas have demonstrated weight loss of 2–3 kg [18,20]. Withdrawals from exenatide trials ranged from 24–30% compared with 9–14% with liraglutide in this study. Nausea with exenatide ranged from 39% with 5 µg to 51% with 10 µg [17] compared with 10.5% for liraglutide. Furthermore, 41% were positive for anti-exenatide antibodies compared with 9–13% with anti-liraglutide antibodies.

With sitagliptin 100 mg once daily for 24 weeks, HbA_1c_ decreased by 0.3% from baseline in subjects receiving glimepiride, with 11% achieving an HbA_1c_ < 7.0%[21]. Reductions in FPG and PPG from baseline were 0.05 and 1.4 mmol/l, respectively, while weight increased by 0.8 kg and the prevalence of nausea was < 1%.

Although head-to-head trials are required to test true differences between these agents, the marked effects of liraglutide on FPG may be as a result of consistent blood levels of liraglutide maintained over 24 h compared with exenatide which has to be administered 60 min before breakfast and dinner and has a half-life of 1.5–3.6 h [22]. In a recent 26-week head-to-head trial comparing liraglutide with exenatide, liraglutide produced a 0.3% greater decrease on HbA_1c_ (*P* < 0.0001) [20]. Because DPP-4 inhibitors inhibit the degradation of GLP-1, the efficacy of sitagliptin is dependent on levels of endogenous GLP-1 which is physiologically low compared with the much higher pharmacological levels of liraglutide. Pharmacological levels may be needed to induce satiety, weight loss and possibly larger HbA_1c_ reductions.

Liraglutide is an effective and well-tolerated once-daily human GLP-1 analogue that improves overall glycaemic control and indices of pancreatic B-cell function with minimal weight gain and risk of hypoglycaemia when used in combination with a sulphonylurea for T2D.

## Competing interests

The study was funded by Novo Nordisk, the manufacturer of liraglutide. In collaboration with the investigators, Novo Nordisk was responsible for the study design, protocol, statistical analysis plans, oversight, analysis and reporting of the results. Data were recorded at the clinical centres and maintained by the sponsor. The LEAD-1 SU study group had full access to the data. Final responsibility for the decision to submit the manuscript for publication was the authors. MM has received lecture fees from Novo Nordisk, Servier, MSD; JS has received honoraria, grants and lecture fees from Novo Nordisk; MB, WMWB and NAK have no conflicts to declare; JS has received lecture fees from Novo Nordisk; MZ is employed by, and holds stock in, Novo Nordisk; TLT is employed by Novo Nordisk; SC is a member of the international advisory board on liraglutide for Novo Nordisk and has received lecture fees from Novo Nordisk.

## Principal investigators for the LEAD-1 SU study group

The principal investigators for the LEAD-1 SU study group were: De Loredo L, Waitman J, Litwak L, Rodriguez M, Vines G, Maffei L, Issa C, Lowy A, Bartley P, Kamp M, Shaw J, Russell A, Karrasch J, Colagiuri S, Karrasch J, Lowy A, Protich M, Hristov V, Hristozov K, Petrova-Gancheva A, Pavlova M, Petkova M, Kokic S, Bacun T, Balen MJ, Perusicova J, Pelikanova T, Smahelova A, Rusavy Z, Zackova V, Honka M, Strand J, Kuusisto A, Honkasalo M, Taskinen M, Ilvesmaki V, Piippo T, Rahtu M, Eriksson J, Kallioniemi V, Korsoff P, Galtier F, Gouet D, Bauduceau, Lorcy Y, Duevezin-Caubet P, Courreges J-P, Le Devehat C, Marre M, Chow F, Bhansali A, Chandalia HB, Seshadri KG, Shah S, Raz I, Cohen O, Karnieli E, Pontiroli A, Santeusanio F, Squatrito S, Ho Yoon K, Hyun Baik S, Taek Woo J, Bebakar WW, Kamaruddin NA, Mumtaz M, Que T, Sy RA, Panelo A, Racho V, Bednarczyk-Kaluzny M, Biniszkiewicz T, Derezinski T, Filipczak R, Gaczarek-Belczyk E, Gumprecht J, Kuleta J, Lopatynski J, Majkowska L, Mader P, Miarka J, Mikolajczyk-Swatko A, Skokowska E, Wojciechowska M, Wolnik B, Serban V, Mogos T, Albota A, Pop L, Popa B, Moore R, Mitha E, Latiff G, Bhana S, Lakha D, Brändle M, Bach-Kliegel B, Lehmann R, Christ E, Gaillard R, Chuang L-M, Tu S-T, Chen J-F, Kuo S-W, Deerochanawong C, Lauhawatana S, Kittivat N, Arslan M, Balci MK, Sargin H, Comlekci A, Karsidag K, Dayan A.

## Abbreviations

AACE: American Association of Clinical Endocrinologists
ADA: American Diabetes Association
AEs: adverse events
BMI: body mass index
CI: confidence interval
DPP-4: dipeptidyl peptidase-4
EASD: European Association for the Study of Diabetes
ECG: electrocardiogram
FPG: fasting plasma glucose
GLP-1: glucagon-like peptide-1
HbA_1c_: glycated haemoglobin
HOMA: homeostasis model assessment
IDF: International Diabetes Federation
IR: insulin resistance
LEAD: Liraglutide Effect and Action in Diabetes
OGLA: oral glucose-lowering agent
PG: plasma glucose
PPG: postprandial plasma glucose
T2D: Type 2 diabetes
